# Keeping an Eye on Future: Medical Tourism

**DOI:** 10.4103/0970-0218.69247

**Published:** 2010-07

**Authors:** R Jose, Sandeep Sachdeva

**Affiliations:** Directorate General of Health Services, Ministry of Health and Family Welfare, Government of India, Nirman Bhawan, New Delhi – 110 108, India

Tourism has been an instrument of social, cultural, religious, and economic phenomena throughout the globe since ancient time. Progress in opportunities of travel, stay, and information and communication technology has further facilitated people moving from one place to another for leisure, business, education, or medical purpose. A booming economy has far-reaching consequences on all sectors of human development including health. This is true for countries in transition especially India.

International tourist arrival throughout the globe was 922 million and India’s share of foreign tourist arrivals (FTAs) stood at 5.4 million during 2008 whereas it was 2.38 million in 2002.(1) Though the growth in overall tourism in India has been impressive with the contribution of 5.9% of GDP (2003–2004) and projected to rise to 10% in the nation’s wealth, India’s share in current global tourist arrivals is quite insignificant (0.58%).(2) Tourism is the third largest net earner of foreign exchange next to garments, gem, and jewelry industry, recording earnings of US $11.7 billion in 2008 (Rs. 50730 crores).(3)

The International Passenger Survey (2003) estimates that about 2.2% of the foreign travelers and 10% of the non-resident Indians (NRIs) visited India with the objective of health care and treatment.(4) India treated approximately 150,000 and 450,000 foreign patients during 2004 and 2007, respectively. According to a study carried under the aegis of Confederation of Indian Industry (CII), medical tourism in India is projected to become a US $2.3 billion industry with an annual growth rate of 30% in 2012 from 12% (2002).(5)

Medical tourism (MT) involves traveling to an advanced facility in another country for receiving requisite medical/surgical treatment at a much lower cost that is often linked with using the savings to opt for and enjoy a holiday during recovery. However, this is largely true for ambulatory/elective interventions. Some of the favored destinations for MT across the globe include Cuba, Costa Rica, Hungary, Israel, Jordon, Lithuania, Belgium, South Africa, Singapore, Thailand, Philippines, Malaysia, India, and South Korea.(6) MT is not a new concept though marketing for the same is of recent origin involving venture between the health care provider and hospitality sector.

Due to aggressive promotion from stakeholders, Indian private hospitals are increasingly finding a mention in the travel itineraries of foreigners. MT is witnessing an upward trend because India has large number of world heritage monuments, archeological and religious sites, and diverse cultural festivals at one end to state-of-the-art infrastructure and skilled medical manpower that is using innovative technology; quality care of service with high success rate/outcome; compassionate nursing care, English-speaking staff, no waiting time for surgeries; and the most critical element of service delivery at a very affordable cost in comparison to well-developed nations. The indicative cost is shown in Table 1.(7)

**Table 1:** Cost comparison of selective interventions (US$)(7)

Country	Procedure (US$)				
	Heart bypass	Hip replacement	Knee replacement	Face lift
India	7000	7020	9200	4800
USA	133,000	57,000	53,000	16,000
Thailand	22,000	12,700	11,500	5000
Singapore	16,300	1200	9600	7500
Malaysia	12,000	7500	12,000	6400
South Korea	31,700	10,600	11,800	6600
Mexico	27,000	13,900	14,900	11,300
Costa Rica	24,100	11,400	10,700	4900
UAE	40,900	46,000	40,200	N/A

On the similar front, outsourcing of diagnostics, promotion of research and development (R&D), rapid growth of research institutes, well-established pharmaceutical industries, initiation of large number of clinical trials, development of newer concepts like surrogate mothers, preservation of cord blood/stem cells, milk banks, and successful use of telemedicine at various settings are some of the catalyzing factors ensuring high visibility and prominence of the Indian health sector in the global news. The rise of Sports Medicine as a subspecialty and successful culmination of Commonwealth Games in New Delhi during the latter half of 2010 will further propagate the phenomenon of MT by leaps and bounds. In addition to above, other value-added service offered in India includes a bouquet of preventive check-ups, wellness therapies, yoga, meditation/spiritual enlightenment, hydrotherapy, mud-therapy, and ayurvedic healing, thus enlarging the scope from “medical” to “health” tourism.

Tourism is overwhelmingly an industry of private sector although public sector has a significant role to play at different levels including the promotion of public private partnership (PPP). Government of India (GoI) has recognized the economic potential of MT and is promoting this underexplored arena through its market development assistance (MDA) scheme especially for accredited institutions to strengthen the Indian health care brand overseas. Under this, hospital groups will be provided financial assistance for publicity through printed materials, travel and stay expenses for sales-cum-study tours, and participation fees for trade fairs and exhibitions. Calendars of international health events are being planned and the events are being held annually. Efforts are being made for introducing uniform pricing band through various consultative processes by stimulating stakeholders for the formation of common platform/consortium. GoI has introduced medical visa for patients desirous of undergoing treatment in India.

On the regulation of human resource for health, Planning Commission (GoI) is encouraging all State Councils to shift to a system of periodical renewal of registration of medical personnel. In the field of paramedical education including physiotherapy and occupational therapy, priority is being given to the establishment of National Paramedical Council as an apex body to determine standards of education/training and to ensure uniform enforcement on the lines of Medical Council of India (MCI) for MBBS/higher courses.(8) In the recent past, GoI has promulgated legislations like “The Clinical Establishment (Registration and Regulation) Bill, 2007” and “The Transplantation of Human Organs (Amendment) Rules, 2008” and has finalized the standard course curriculum of 20 paramedical courses (2009) in the country. Such has been the magnitude of confidence on MT that private institution(s) in India have started a 2-year health management course with specialization in MT. These measures will have a long-term and/or in-direct impact on the MT sector.

To enhance visibility and scalability and attract more clients, health institutions in India will require intensive efforts on the establishment and compliance of rigorous quality standards, implementation of international best practices, and communication thereof. Health services are increasingly coming under independent evaluation by accreditation agencies in many countries. Accreditation is a voluntary process by which an authorized agency/organization evaluates and accredits health service according to a set of standards describing the structure and processes that contribute to desirable patient outcomes. The patients get services by credential medical staff. Rights of patients are respected/protected and regularly evaluated through patient satisfaction and simultaneously there is an element for professional growth/enrichment.

Accredited institutions are potential winners in attracting and retaining foreign clients. Some private health institutions in India have taken up accreditation from JCI(9) and still significant proportion on ISO: 9001-2000 certification through their own efforts. National Accreditation Board for Hospitals and Health Care Providers (NABH) and National Accreditation Board for Testing and Calibration Laboratories (NABL), a constituent Board of Quality Council of India, have adopted standards and accreditation process in-line with worldwide accreditation practices and many of Indian hospitals/laboratories/diagnostic centers in the recent past have initiated, applied, or received their accreditation.(10)

In spite of very optimistic, supportive, and economically driven environment being garnered for MT, some of the issues and concerns could be incentives, concessions and taxation, follow-up services, limited presence of global health insurers, exodus of specialists from public health system to private sector, malpractices by patients/attendants, litigation, legal stay, and import of exogenous infection(s). However, taking a clue from the current scenario, it would be apt to conclude that MT has a huge potential in times to come as more and more foreign travelers will be visiting India for their health needs and keeping an eye on future will lead to quantitative and qualitative gains for both international and national clients.
